# Efficient Genetic Method for Establishing *Drosophila* Cell Lines Unlocks the Potential to Create Lines of Specific Genotypes

**DOI:** 10.1371/journal.pgen.1000142

**Published:** 2008-08-01

**Authors:** Amanda Simcox, Sayan Mitra, Sharon Truesdell, Litty Paul, Ting Chen, Jonathan P. Butchar, Steven Justiniano

**Affiliations:** Department of Molecular Genetics, Ohio State University, Columbus, Ohio, United States of America; Stowers Institute for Medical Research, United States of America

## Abstract

Analysis of cells in culture has made substantial contributions to biological research. The versatility and scale of *in vitro* manipulation and new applications such as high-throughput gene silencing screens ensure the continued importance of cell-culture studies. In comparison to mammalian systems, *Drosophila* cell culture is underdeveloped, primarily because there is no general genetic method for deriving new cell lines. Here we found expression of the conserved oncogene *Ras^V12^* (a constitutively activated form of *Ras*) profoundly influences the development of primary cultures derived from embryos. The cultures become confluent in about three weeks and can be passaged with great success. The lines have undergone more than 90 population doublings and therefore constitute continuous cell lines. Most lines are composed of spindle-shaped cells of mesodermal type. We tested the use of the method for deriving *Drosophila* cell lines of a specific genotype by establishing cultures from embryos in which the *warts* (*wts*) tumor suppressor gene was targeted. We successfully created several cell lines and found that these differ from controls because they are primarily polyploid. This phenotype likely reflects the known role for the mammalian *wts* counterparts in the tetraploidy checkpoint. We conclude that expression of *Ras^V12^* is a powerful genetic mechanism to promote proliferation in *Drosophila* primary culture cells and serves as an efficient means to generate continuous cell lines of a given genotype.

## Author Summary

In *Drosophila*, the genetic analysis of whole animals has been the focus of the field and has been exceptionally successful. Gene discoveries in flies have led to parallel studies in vertebrates and hence have accelerated the understanding of biology. Furthermore, some 60–70% of human disease genes are conserved in *Drosophila*, thus making the genetically tractable fly a useful disease model. While the whole-organism approach in *Drosophila* is powerful, there are studies that can best be conducted in cell lines. In this regard, *Drosophila* lags far behind mammalian systems, in which creation of cell lines using genetic manipulation is routine. We sought to test whether similar genetic approaches could be used in *Drosophila*. We discovered a simple genetic method for the rapid production of fly cell lines using an activated oncogene to stimulate proliferation in cultured embryonic cells. The method has immediate application for creating custom cell lines of a given genotype. We provided an example of this by making lines in which a tumor suppressor gene is targeted. Specifically designed cell lines will be extremely valuable for gene discovery using whole-genome RNAi screens and for producing large numbers of cells of a specific genotype for biochemical studies.

## Introduction

Mammalian somatic-cell tissue culture has a long history that has led to the sophisticated approaches available today for making cell lines from various cell types and genetic backgrounds. In comparison with mammalian systems, *Drosophila* somatic-cell culture is in its infancy [1]. *Drosophila* cell lines are commonly derived spontaneously from primary cultures of embryos and the process of generating a line is often protracted (for example, [2]–[5]). The problem stems from the fact that nothing is known about genetic changes which presumably underlie the ability of the cells to proliferate indefinitely. There is great interest in developing lines derived from particular genotypes or cell types for biochemical studies and for high throughput screens utilizing gene silencing [6]. A recent report describes the generation of germ cell and somatic stem cell lines from *Drosophila* ovaries, which are mutant for the tumor suppressor *bag of marbles* [7]. This suggests genetic approaches that increase a given cell population and/or genetic changes that influence cell proliferation may assist in the development of *Drosophila* cell lines.

By analogy with vertebrates, *Drosophila* cells could be immortalized and transformed through repression of tumor suppressor genes and activity of oncogenes. In mammalian systems, a common approach to generating immortal cells is to supply telomerase and inhibit the tumor suppressors Rb/p53 with large T antigen. Transformed phenotypes can then be induced by expression of oncogenes such as Myc and activated Ras. Multiple tumor suppressor genes have been identified in *Drosophila* through their ability to produce abnormal growth *in vivo* (reviewed in [8],[9]). Similarly, activated Ras can cause hyperplasia in *Drosophila* [10]. Activated Ras promotes growth and cell cycle progression by increasing the levels of Myc and PI3K signaling [11],[12]. These *in vivo* phenotypes manifest as outgrowths of imaginal tissue suggesting that changing the activity of tumor suppressors or oncogenes has the potential to also alter cell proliferation *in vitro*.

Here we tested the effects of Ras *in vitro*, by expressing a constitutively activated form, Ras^V12^, in *Drosophila* primary cultures. Expression of *Ras^V12^* caused dramatic changes in cell proliferation and we have found that it provides a method to efficiently develop new cell lines. This is a significant advance in *Drosophila* tissue culture that will be immediately valuable for generating cells of specific genotypes, and with further development may also be used for creating tissue-specific cell lines.

## Results

### Expression of Ras^V12^, but not Myc, in Primary Cultures Promotes Cell Proliferation

To determine the effects of oncogene expression in *Drosophila* tissue-culture cells, we established primary cultures from embryos in which Ras^V12^ (an activated form of Ras locked in the GTP-bound state) or Myc could be induced in single cells and inherited in clonal derivatives using the flip-out technique [11]–[13]. The cells were heat shocked to induce single cells to express UAS-regulated oncogenes and the cell marker green fluorescent protein (GFP) under the control of *Act5C-GAL4*. Act5C is a cytoplasmic actin and drives GAL4, and consequently UAS-transgene, expression in many cell types. Cells in control cultures were induced to express GFP alone.

**Figure 1:**
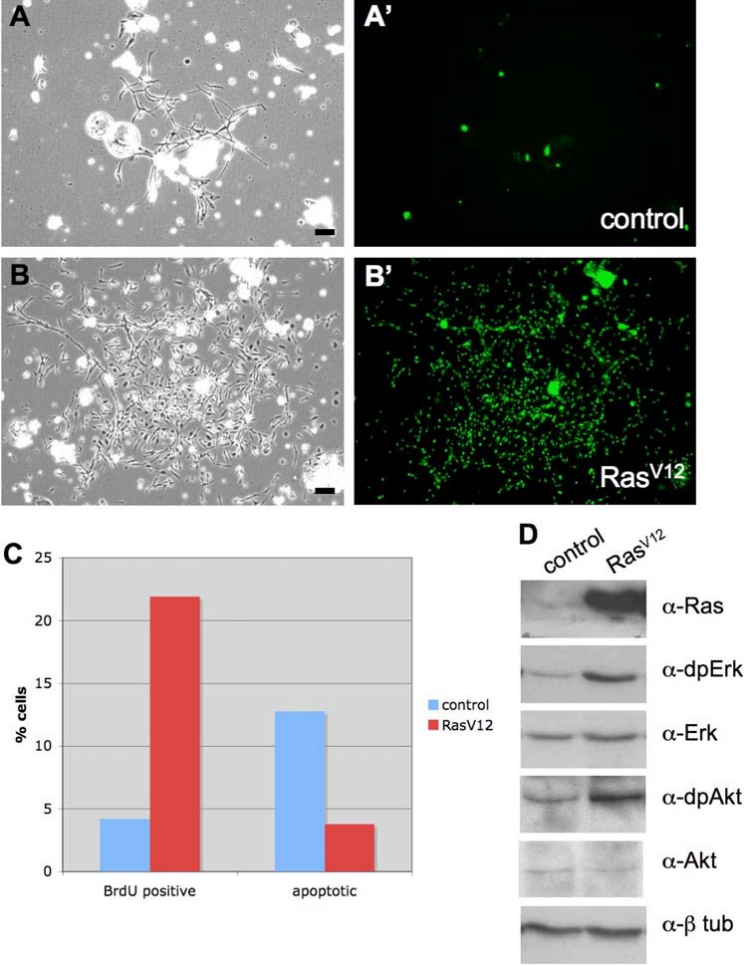
Expression of *Ras^V12^* promotes cell proliferation *in vitro*. The FLP-FRT system was used to generate clones of marked cells expressing GFP alone or in combination with the *Ras^V12^* oncogene. (A–B) phase images of cells and (A′–B′) corresponding GFP images. (A) Control culture showing a small patch of fibroblast-like cells. (A′) The fibroblast-like cells are GFP-, only single and pairs of round cells are GFP+ *(Act5C-GAL4; UAS-GFP)*. (B) *Ras^V12^*–expressing culture showing large patch of fibroblast-like cells. (B′) The cells are GFP+ and comprise a clone *(Act5C-GAL4; UAS-GFP, UAS-Ras^V12^)*. All clones shown are 10 days following induction. (C) Fluorescent activated cell sorting (FACS) analysis was used to determine the number of cells in S-Phase (BrdU incorporation) and undergoing apoptosis. More *Ras^V12^* cells were in S-phase and fewer were apoptotic. Both these factors contribute to the larger clone size observed (see A and B above). (D) Control and *Ras^V12^* -expressing primary cultures were analyzed for expression of Ras, dpErk (the phosphorylated active form of Erk, which is generated by signaling through Ras) and pAkt (the phosphorylated active form of Akt, which is generated by signaling through PI3K). Higher levels of Ras, dpErk and pAkt were found in the *Ras^V12^* -expressing cells. Erk, Akt and β-tubulin were used for loading controls. (A–B, Scale bar, 50 µm).

Ten days after induction of *UAS-GFP* in control cultures there were very few clones of GFP-expressing cells comprising more than a few cells (Figure 1A). Rare patches of spindle-shaped cells were observed but these were not all GFP-positive clonal derivatives of a single cell (Figure 1A). There was a dramatic difference in the *Ras^V12^*-expressing cultures. Ten days after induction of *UAS-Ras^V12^*, there were numerous large clones of GFP-expressing cells (Figure 1B). Most clones were comprised of spindle-shaped cells. In 3–4 weeks the cultures were confluent with GFP positive Ras^V12^-expressing cells. At this time the control cultures were still dominated by differentiated cell types and only small clones of GFP positive cells.

In contrast to Ras^V12^, expression of the Myc oncogene did not produce large clones of cells. Very few cells expressing GFP/Myc were observed (not shown). Simultaneous expression of Ras^V12^ and Myc, however, did result in large clones of cells and the primary cultures followed a similar course as those expressing Ras^V12^ alone, reaching confluence in about 3–4 weeks (not shown).

In primary cultures expressing Ras^V12^, the fraction of cells in S-phase was elevated compared with controls and fewer cells died by apoptosis suggesting that both an increase in cell proliferation and reduction in cell death contribute to the larger clone size (Figure 1C). Expression of Ras^V12^ increased activity of the MAPK/Erk pathway, which is the canonical route of Ras signaling in *Drosophila* (Figure 1D). Akt phosphorylation was also enhanced, consistent with the activation of PI3K signaling that has been observed for this oncogenic form of Ras *in vivo* (Figure 1D; [12]).

### Cell types expressing *Ras^V12^* in primary cultures

**Figure 2:**
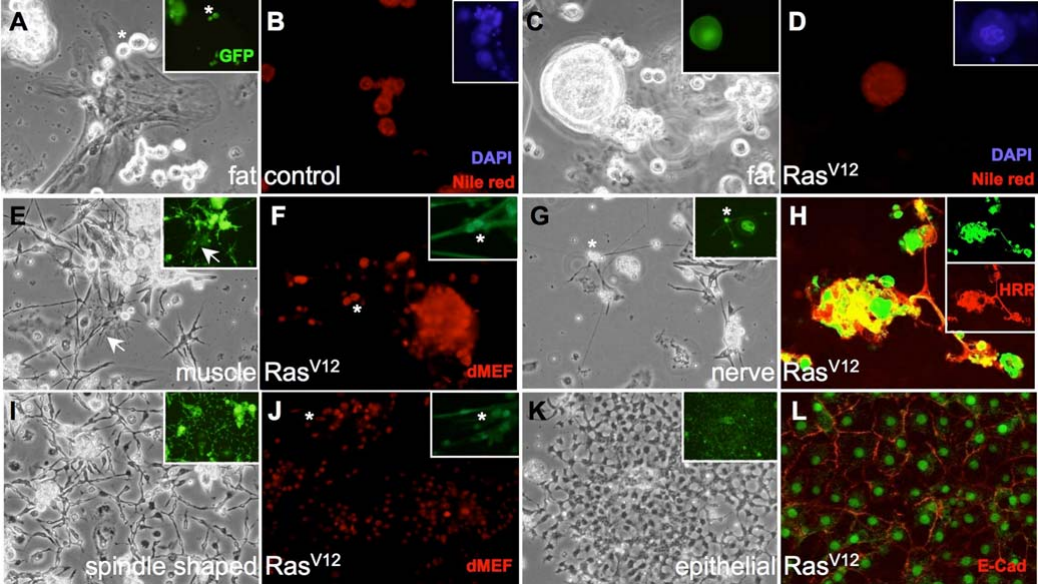
Cell types in *Ras^V12^*-expressing primary cultures. All images except where noted are *Ras^V12^*-expressing cells *(Act5C-GAL4; UAS-GFP, UAS-Ras^V12^)*. (A) Control fat cells expressing GFP (*)*(Act5C-GAL4; UAS-GFP)* are a similar size to GFP- cells. (B) Control cells stained for fat (Nile red), the inset shows nuclei stained with DAPI. (C) *Ras^V12^*-expressing fat cell is greatly enlarged (GFP+) compared to control cells (GFP-). (D) *Ras^V12^*-expressing fat cell stained with Nile red and DAPI (inset). The nucleus is enlarged due to endoreplication (compare with inset in (B)). (E) *Ras^V12^*-expressing muscle cells (arrow). These cells actively twitch. (F) *Ras^V12^*-expressing muscle cells express the mesodermal marker dMef2. The inset shows the detail of a muscle cell with two nuclei (*). (G) Ras^V12^-expressing nerve cells with axons. The inset shows a detail of the axons (*). (H) Confocal image of control and Ras^V12^-expressing (GFP+) nerve cells (HRP+). Both genotypes are present in the clump of cell bodies and axon bundle. (I) Spindle-shaped Ras^V12^-expressing cells, which are the most common proliferating cell type and predominate the culture. The cells are typically bi-polar but a range of morphologies are seen with different length processes. (J) The spindle shaped Ras^V12^ cells express dMef2. (K) Epithelial-like Ras^V12^-expressing cells. The cells form a flat sheet. (L) Confocal image of Ras^V12^ cell sheet expressing the epithelial marker E-Cadherin at the cell periphery.

Similar types of cells developed in primary cultures derived from all genotypes. After 10 days in culture, these included fat, muscle, nerve, blood, spindle-shaped, and epithelial cells, which are typical of *Drosophila* primary cultures and can be recognized by their distinct morphologies (Figure 2) [14]–[16]. We confirmed cell type by using specific stains and antibodies (Figure 2). Fat cells in both Myc- and Ras^V12^-expressing cultures were very large as a result of endoreplication (Figure 2A–D; Figure S1). The size of the Ras^V12^-expressing cells was consistently much larger than the Myc-expressing cells (Figure S1). A role for *Drosophila* Myc in endoreplication has also been shown *in vivo* [17]–[19], but this has not been reported for Ras. Control and *Ras^V12^-*expressing muscle and nerve cells were common (Figure 2E–H). We used a pan-hemocyte antibody to detect blood cells [20]. These cells were rare in early primary cultures of all genotypes and only occurred in a subset of older cultures (not shown). The sporadic development of blood in primary cultures has been noted [16]. The most predominant cell types expressing *Ras^V12^* were spindle-shaped and epithelial cells (Figure 2I–L). These cells types were rare in control cultures. The spindle-shaped cells, which comprised the single most dominant cell type, expressed the mesodermal marker dMef2 (Figure 2J; [21]). The epithelial-like cells, which formed flat cell sheets, expressed the epithelial marker, E-Cadherin (Figure 2L). Somewhat surprisingly, these epithelial cells also expressed dMef2 (not shown). However, there are known instances of epithelial dMef2 expression *in vivo*; the ovarian follicle cells, which form an epithelium covering the developing oocyte, are known to express dMef2 [22].

### *Ras^V12^*-Expressing Cells Give Rise to a Cell Population that can be Passaged for Prolonged Periods and Appear Immortalized and Transformed

**Figure 3:**
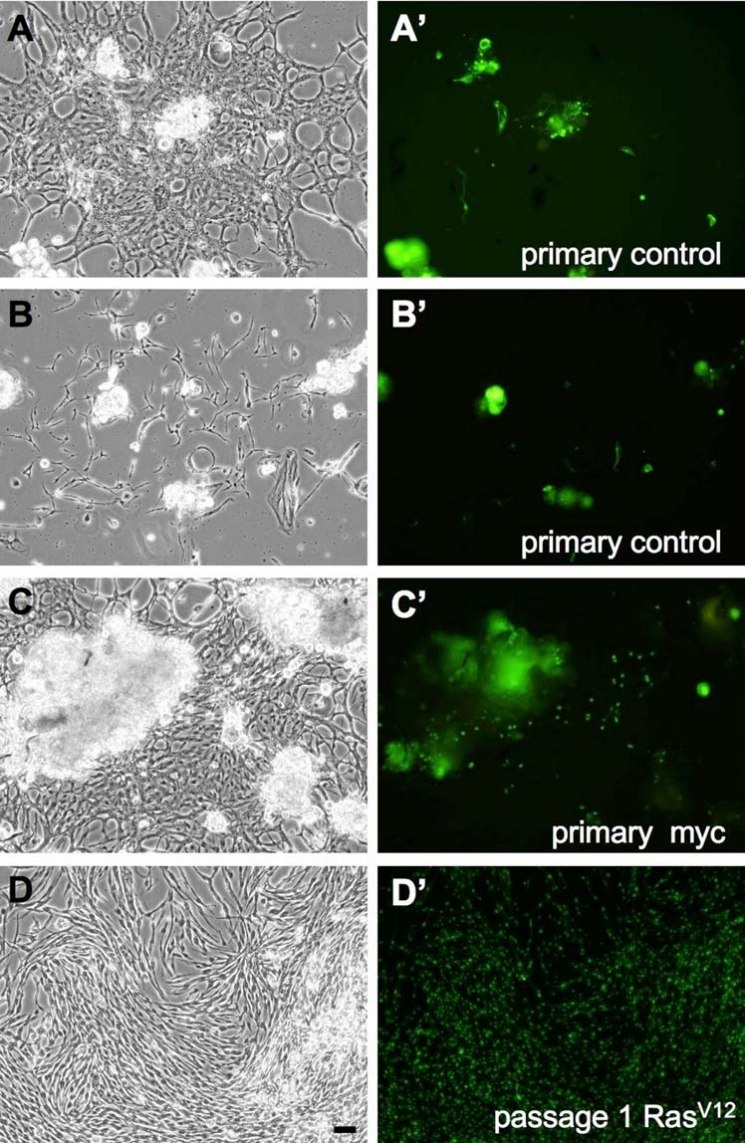
*Ras^V12^*-expression reduces the time for cultures to reach confluence and increases the success of passaging. (A–D) phase images of cells and (A′–D′) corresponding GFP images. All images are from 10 weeks after establishment of primary cultures. (A–B′) Examples of primary control cultures showing patches of fibroblast-like cells. The culture is not yet confluent and only scattered cells are GFP+. (C) Myc-expressing primary culture. The fibroblast-like cells comprising most of the culture are control cells not expressing Myc. Scattered single cells and some cells in amorphous clumps are Myc, GFP+. These amorphous clumps of neural were seen in cultures of all genotypes. D) *Ras^V12^*-expressing cells from the first passage. By 10 weeks, *Ras^V12^*-expressing primary cultures have grown to confluence and have already been passaged. (Scale bar, 50 µm).

In order to determine if expression of Ras^V12^ would facilitate the establishment of *Drosophila* cell lines, we set up cultures from embryos in which the cells expressed *UAS-Ras^V12^* directly under the control of the broadly expressed *Act5C-GAL4* gene. The cultures were maintained for the long term and passaged when they reached confluence. In parallel, we established cultures from controls, Myc-, and Ras^V12^; Myc-expressing embryos. We found that expression of Ras^V12^ accelerated the time to the first passage to about 3 weeks, whereas, controls could only be passaged for the first time after 16–29 weeks (Table 1, Figure 3). Moreover, all Ras^V12^-expressing cultures could be passaged multiple times and established as continuous lines. Most have now undergone more than 60 passages, which is an equivalent of about 120–240 population doublings. One half of the control cultures grew sufficiently well to be passaged at least once, however, only 3 (of 27 total) continued to proliferate (Table 1). A success rate of cell line establishment from about one of ten primary cultures is typical for *Drosophila* embryos [2]. Myc expressing cells rarely survived in culture and did not achieve sufficient density to be passaged (Figure 3C), but cells expressing Myc and Ras^V12^ could be passaged and established as lines (Table 1).

**Table 1:** Summary of primary culture development.

Genotype (n primary cultures)	Weeks to confluence (n primary cultures)	Months to passage 10 (n lines)
Control (27)	16–29 (16)	12–18 (3)
Ras^V12^ (11)	3 (11)	5–8 (11)
Myc (14)	NA*	NA*
Ras^V12^ Myc (9)	5–6 (9)	6–9 (9)
Ras^V12^ wts^RNAi^ (8)	2–3 (8)	6–10 (7)
wts^RNAi^ (9)	8–11 (9)	11–15 (4)

*: NA not applicable. Myc expressing cells did not proliferate sufficiently well to reach confluence or be passaged.

**Figure 4:**
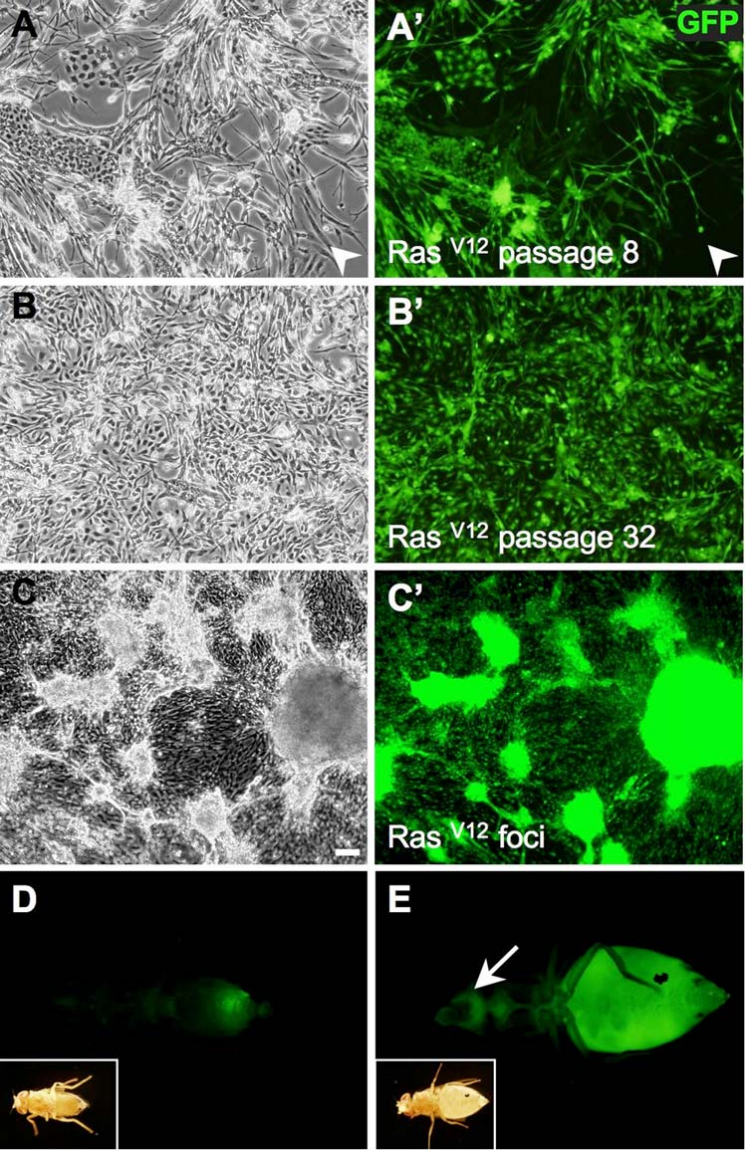
Properties of *Ras^V12^*-expressing cell cultures. (A–C) Phase contrast images. (A′–C′, D–E) GFP images. (A, A′) *Ras^V12^*-line 7 at passage 8. There are a number of different cell morphologies and levels of GFP expression. Some cells do not express GFP (arrowhead). (B, B′) *Ras^V12^*-line 7 at passage 32. The cells are more homogeneous in morphology and GFP expression levels. (C, C′) *Ras^V12^*- expressing cells form foci characteristic of transformed cells. (D) Fly injected with *Ras^V12^* cells on day 0. (E) Fly on day 7 after injection with Ras^V12^ cells. The tumor cells have migrated to distant sites including the head (arrow). In (D and E) the insets show a bright field image of the injected fly. (Scale bar (C), 50 µm in A–C).

In early passages, the Ras^V12^-expressing cultures had heterogeneous cell morphologies and varying levels of GFP expression and even included some cells that were Ras^V12^/GFP negative (Figure 4A). This variety of cell types suggests an oligoclonal origin of the cultures. In early passages cells took longer to grow to confluence and growth was not uniform across the flask suggesting some cells grew more efficiently in culture. In later passages, however, the cells appeared more homogeneous, suggesting a single or a few cell types predominated (Figure 4B). There was more variation in the levels of Ras expression in independent cell lines (1.0 to 3.6 fold; Figure S2A) than in the evolution of a single line (1.0–1.3 fold; Figure S2B).

The extended growth in culture suggests the cells are immortal. Most lines also show features of transformation. The cells are not contact inhibited or density dependent and can grow piled up in foci (Figure 4C). We also tested whether the cells were able to form tumors in flies. Ras^V12^/GFP-expressing cells were injected into the abdominal cavity of females. After 7–10 days these hosts died and Ras^V12^/GFP positive cells were observed as far distant from the injection site as the head (Figure 4D and E).

**Figure 5:**
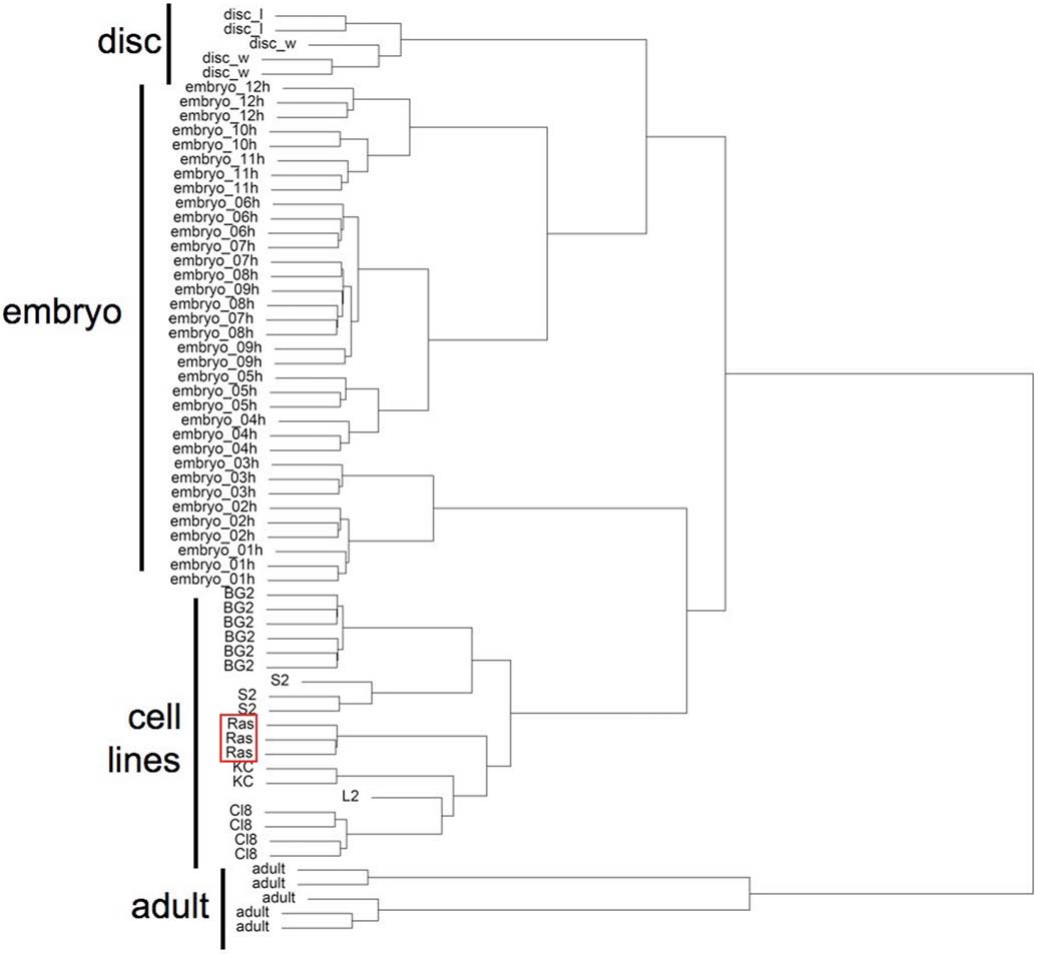
*Ras^V12^*-expressing cells share a transcriptional signature with established cell lines. Cluster analysis of microarray expression data groups Ras^V12^ line 11 cells (boxed) with other cell lines (cell line names given) and away from *in vivo* samples; adults, embryos (embryo, stage in hours) and imaginal discs (leg, l, wing, w). The top 20% of transcripts ranked by standard deviation were used to generate the dendogram.

Further support that the Ras^V12^-expressing cells represent bona fide continuous cell lines is provided by their genome-wide transcriptional profile. By analyzing microarray datasets from embryos, adults and established cell lines, we defined a set of genes that are differentially expressed in tissue-culture cells versus *in vivo* tissues (Butchar et al. in preparation, Figure S3). Ras^V12^ cells (line 11) clustered very closely with the established cell lines because they had a similar expression pattern (Figure 5).

### Establishment of Custom Cell Lines

The strategy we have developed will allow the efficient production of cell lines carrying a mutation or transgene of interest. To demonstrate this we established cell cultures in which the *warts (wts)* tumor suppressor gene [23],[24] is silenced by RNAi. Primary cultures were established from embryos expressing *UAS-Ras^V12^* and *UAS-wts^RNAi^* transgenes. The cells could be subcultured in about 3 weeks and a number of continuous lines were established (Table 1). Quantitative PCR showed that *wts* mRNA levels were reduced to between 10% and 75% of the control cell level in the 6 *UAS-Ras^V12^; UAS-wts^RNAi^* lines (Figure S4A). We also tested the transgene *in vivo* and found the *wts^RNAi^* phenotype closely resembled that of a *wts* mutant, causing tumors and organ size enlargement (Figure S4B–E).

**Figure 6:**
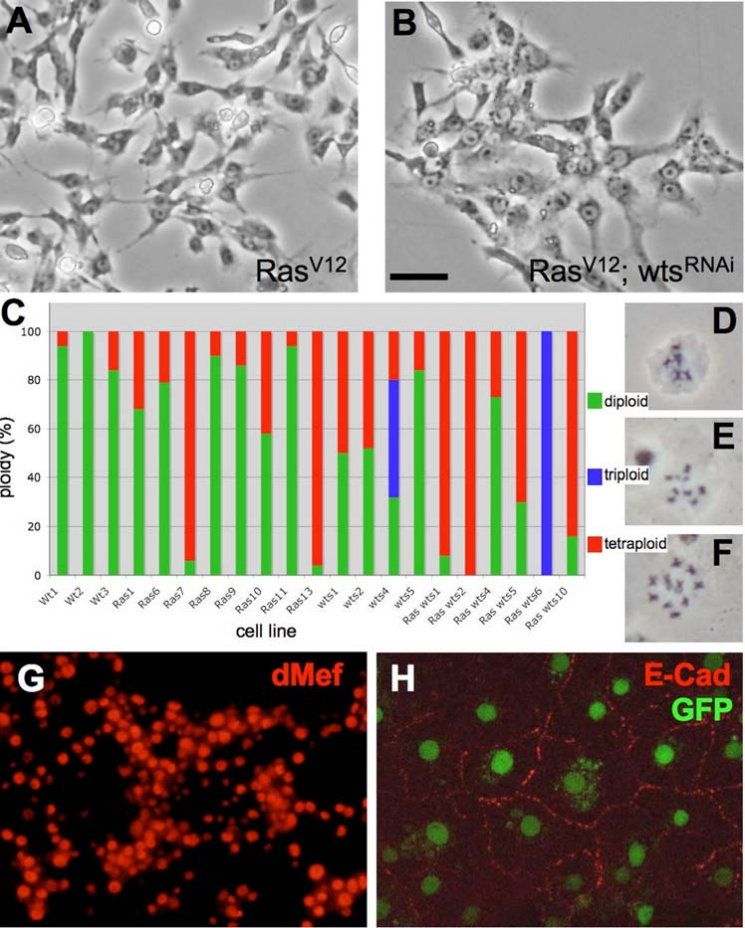
Use of *Ras^V12^* expression to generate cell lines expressing a *wts^RNAi^* transgene. The *Ras^V12^ wts^RNAi^* cells are larger than *Ras^V12^* cells and primarily tetraploid. (A) Ras^V12^ cells from line 11, which are predominantly diploid (94%). (B) Cells from Ras^V12^ wts^RNAi^ line 10, which are predominantly tetraploid (84%) and relatively large (compare cell size in A and B). (C) Histogram showing ploidy of various cell lines (green, % diploid; blue, % triploid; red, % tetraploid). *Ras^V12^ wts^RNAi^* cells are significantly more polyploid than wild type (p = 0.001) and *Ras^V12^* cells (p = 0.007). (D–F) Chromosome spreads of diploid, triploid, and tetraploid cells, respectively. The small 4^th^ chromosome is often lost in cells in culture and/or not visible in karyotype spreads. (G) *Ras^V12^* -line 10 expresses dMef suggesting it is of mesodermal origin. (H) Confocal image of Ras^V12^; wts^RNAi^ cells. The cells have an epithelial-like morphology and expresses E-Cadherin. (Scale bar (B), 50 µm in A and B).

In general, the *Ras^V12^*; *wts^RNAi^* cells appeared larger than cells expressing *Ras^V12^* alone (Figure 6A and B). Large size is often associated with increased DNA content and we examined the ploidy of the lines. We determined the fraction of cells in a given line that were diploid, triploid or tetraploid (Figure 6C–F). We found most of the *Ras^V12^*; *wts^RNAi^* lines (4/6) were predominantly tetraploid, one was triploid, and one was 25% tetraploid (Figure 6C). In contrast, the 3 wild-type cell lines generated in this study were predominantly diploid, as were 6/8 cell lines expressing *Ras^V12^* alone (Figure 6C). We also established 4 cell lines expressing a *wts^RNAi^* transgene (Table 1). Inhibiting *wts* expression did promote the formation of cell lines; about 1 in 2 progressed to continuous lines compared with 1 in 10 for wild-type cultures (Table 1). However, these took longer to establish than those expressing *Ras^V12^* (Table 1). One *wts^RNAi^* line is mainly diploid, one is a mixture of diploid, triploid and tetraploid cells, and the others are about 50% tetraploid (Figure 6C). Taken together these data suggest that Ras activation and Wts inhibition leads to changes in ploidy, as *Ras^V12^*; *wts^RNAi^* cells are significantly less diploid than wild type (p = 0.001) or *Ras^V12^* cells (p = 0.007) (Figure 6C). Wts inhibition alone also appears to have an effect, but with the small sample size the difference to wild type was not significant (p = 0.051).

### Cell Types Represented in Cell Lines

Most cell lines were comprised of spindle shaped cells (for example, Ras^V12^ line 7; Figure 4B). One control cell line had a round cell morphology and one *Ras^V12^*; *wts^RNAi^* cell line had an epithelial-like morphology. We surveyed the control, *Ras^V12^*, *wts^RNAi^*, and *Ras^V12^*; *wts^RNAi^* cell lines (Table 1) with cell-specific antibodies to test for the presence of muscle, nerve, blood, and epithelial cells. All cells were positive for dMef suggesting they are of mesodermal origin (for example, *Ras^V12^* line 10; Figure 6G). One cell line of *Ras^V12^*; *wts^RNAi^* genotype was also positive for E-Cadherin and had an epithelial morphology (Figure 6H).

## Discussion

*Drosophila* tissue culture has lagged behind mammalian systems in part because a directed genetic method to derive cell lines is lacking. Here we found that proliferation induced by activated Ras–expression *in vitro* is dramatic and facilitates the rapid production of cell lines. Primary cultures reached confluence in about 3 weeks. Equivalent cell densities were only achieved in a fraction of control cultures and only after a protracted time of about 16–29 weeks. Also in contrast with controls, the *Ras^V12^*-expressing cells could be routinely propagated from these confluent primary cultures. On average *Ras^V12^*-expressing cultures were passaged 10 times (20–40 population doublings) within 5–8 months. Control primary cultures rarely gave rise to continuous lines and took 12–18 months to reach the 10^th^ passage.

The ability of activated Ras to stimulate growth in *Drosophila* primary cells as shown here, and *in vivo* [10], is in striking contrast to its effect on mammalian cells. In primary mammalian cultures and *in vivo*, activation of Ras induces a growth arrest termed oncogene induced senescence (OIS) [25]–[28]. *In vivo*, OIS functions as a block to tumorigenesis and thus is a protective mechanism for the organism. For oncogenic Ras to transform mammalian cells in culture, the cells must already be immortal. Immortal cells have passed through two key transitions, so-called replicative senescence (M1) and crisis (M2) [29]. M1 can be bypassed if checkpoints involving tumor suppressor genes are inactivated. Crisis is avoided in rare cells in which telomerase is reactivated. Both M1 and M2 can be bypassed if cells are supplied with telomerase to maintain telomere length [30].

It is not clear why *Drosophila* primary cells expressing oncogenic Ras^V12^ behave differently than mammalian cells and continue to proliferate. Two possibilities are considered here:

First, the response may reflect the different mechanism by which *Drosophila* cells maintain their telomeres. In flies, there is no telomerase and the ends of chromosomes are maintained by mechanisms involving transposition and recombination of the non-LTR retrotransposons, HeT-A, TART and TAHRE into telomeric regions [31]–[38]. If this activity were not lost overtime, fly cells would not be subject to the senescence that is caused in part by telomere shortening. *Drosophila* cells with extended growth opportunity, such as cells in culture, may therefore, have the potential to be immortal. This is true for imaginal disc cells, which can proliferate for years if they are cultured *in vivo* in adult hosts where hormonal differentiation cues are absent [39]. Thus, at least some cells in primary cultures of *Drosophila* embryos may be functionally immortal and if challenged with an activated oncogene rather than undergoing OIS, they continue to proliferate.

Second, the *Ras^V12^*-expressing cells that continue to proliferate could have acquired additional genetic changes that allow them to pass through the hypothetical fly equivalences of both M1 and M2. By analogy with mammals, mutations in tumor suppressor genes that regulate cell cycle checkpoints are candidates for inactivation and bypass of M1. Given the different mechanism by which flies replicate telomeres (discussed above) it is not clear whether or how or M2 would apply to fly cells.

The growth pattern of the primary cultures is consistent with either/or both of these possibilities: Initially, cultures were slow to proliferate and proliferation was not uniform across the flask, suggesting that the small subset of cells that do proliferate are cells that are already immortal or have acquired additional genetic changes that confer immortality. The relatively short time frame in which to acquire additional mutations, prior to establishing the lines, may favor the first interpretation.

The ability of activated Ras to promote cell line production means that custom lines of specific genotypes can be created. To demonstrate this, we used *Ras^V12^*-expression to generate cell lines that also express a *wts^RNAi^* transgene. We are also in progress of making a cell line from a cell viable null allele of a gene in the Notch pathway. These cells are currently at passage 10, and western analysis shows they lack the corresponding protein, demonstrating the general utility of the method (AS, unpublished).

*wts* is a tumor suppressor gene that functions in the Hippo pathway [23],[24]. Signaling through this conserved pathway regulates cell death and proliferation in flies and mammals and hence contributes to organ size and tumor development [8], [40]–[42]. Interestingly, we found expression of the *wts^RNAi^* transgene is correlated with increased tetraploidy in the cultured cells (Figure 6). In mammals there are two *wts*-related genes, *lats1* and *lats2* and loss of function of the genes is linked to human cancers [43]–[46]. Both have been implicated in functioning in the tetraploidy checkpoint [47]–[49]. As tetraploidy is often a prerequisite for aneuploidy, a hallmark of cancer cells, the roles of *lats1/lats2* in the checkpoint may be linked to their function as tumor suppressors. Our data suggest that this function may be conserved by the fly gene and the *wts^RNAi^* cell lines. This result also exemplifies the importance of analyzing cells in culture in order to reveal phenotypes that are only apparent after extensive opportunity for growth. This may be particularly important when studying the role of fly genes in processes that manifest themselves as somatic diseases in mammals only after a protracted latency period, such as cancers.

While the system to establish cell lines described here has an important and immediate application to derive cells of a given genotype, in the future, it will also be important to develop additional features. First, the control of *Ras^V12^*-expression using, for example, a drug inducible system [50] will allow cells to proliferate in the presence of the drug and *Ras^V12^* expression, but resume a ‘normal’ state when drug is removed and *Ras^V12^* is switched off. The system could also be used to derive cell lines corresponding to specific cell types, by targeting Ras^V12^ expression with cell-specific GAL4 activators. Our data showing *Ras^V12^*-induced proliferation of cells with distinct morphologies in primary cultures and the creation of an epithelial-like cell line suggest this is likely to be possible. However, as with mammalian cells, culture conditions such as substrates and factors may need to be tailored to support growth of specialized cell types. Currently the system described here favors generation of lines with a cell type that is spindle shaped and of mesodermal origin—somewhat analogous to mouse embryonic fibroblasts, which are used extensively for analyzing genetic mutants. Likewise we expect this method will be valuable for generating an *in vitro* source of large numbers of genetically identical mutant fly cells.

## Materials and Methods

### Fly Stocks and Crosses

For clonal analysis, primary cultures were established with embryos from the following crosses. Control: *HS-FLP X Act5C<CD2>GAL4*, *UAS-GFP*. Ras^V12^: *HS-FLP*; *UAS-Ras^V12^ X Act5C<CD2>GAL4*, *UAS-GFP*. Myc: *HS-FLP*; *UAS-Myc X Act5C<CD2>GAL4*, *UAS-GFP*. Ras^V12^/Myc: *HS-FLP*; *UAS-Ras^V12^*, *UAS-Myc X Act5C<CD2>GAL4*, *UAS-GFP*. After 1–3 days in culture (22°C) the cells were subjected to a 30-minute heat shock (37°C) to induce HS-FLP, which removes the FRT flanked cassette (<CD2>) inserted in the *Act5C-GAL4* gene. This makes GAL4 active and able to induce stable expression of the *UAS-transgenes* [13]. For producing long-term cultures, embryos with UAS transgenes under direct control of *Act5C-GAL4* were used *(Act5C-GAL4/TM6 X UAS-GFP*, *UAS-transgene(s))*. In these cultures half the cells express GFP and the transgene being tested.

### Generation of *wts* RNAi Transgene

An 899 bp fragment corresponding to 2604–3503 of a *wts* cDNA, the RNAi ‘trigger’, was cloned into pBlueScript-KS, with an artificial intron from the *vn* gene [51],[52]. This sense strand ‘trigger+intron’ fragment was then cloned into pUAST. The dsRNA construct was completed by adding the trigger fragment in reverse orientation into pUAST containing the ‘trigger+intron’ fragment. Transgenic lines were established and tested by crossing to the *en-GAL4* driver. Phenotypes including tumors in the abdomen and wing overgrowth were seen (Figure S4).

### Establishing Primary Cultures and Passaging Cells

Embryos were collected overnight at 17°C on grape juice plates supplemented with killed yeast paste. Embryos were rinsed from the plates and collected in a sieve. The embryos were transferred to a 15 ml conical tube using TXN (0.7% NaCl, 0.02% Triton X-100). The TXN was replaced with 50% bleach in water for 3–5 minutes to remove the eggshells and surface sterilize the embryos. The embryos were washed extensively with TXN and transferred to a homogenizer (Wheaton 5 ml). The embryos were rinsed once in water and once in 3 ml medium (Schneider's medium, Sigma, supplemented with 10% heat-inactivated fetal bovine serum, and 1/100 dilution of streptomycin penicillin liquid, Invitrogen). The embryos were homogenized in 3 ml medium with 3 gentle strokes. Large cell clumps and unbroken embryos were allowed to settle and the supernatant was removed to a 15 ml conical tube. The remaining embryos and tissue clumps were homogenized in a second aliquot of medium with slightly firmer strokes and the homogenates were combined. The cells were pelleted by centrifugation and rinsed with three changes of medium. The cells were plated in 25 cm^2^ T-flasks and grown at 22°C. Typically, a starting aliquot of approximately 100 µl of packed embryos was seeded into 3 flasks. To maintain the primary cultures, the medium was changed every 2 weeks. Confluent cultures were trypsinized and diluted 1/2–1/4 into new flasks. Early passages were often difficult to establish and slow to grow to confluence. The parent culture was maintained for as long as possible (by supplying fresh medium to the cells that remain after trypsinization) and typically used to establish multiple first passage cultures before one line showed successful continued growth.

### Cell Proliferation Assay

Cells in culture flasks were labeled with bromodeoxyuridine (BrdU; 10 µM) for 4 hours at 22°C. Approximately 1×10^6^ cells were stained with APC conjugated anti-BrdU antibody and propidium iodide (PI, 5 mg/ml) (BD Biosciences protocol, Chicago, IL, USA). Labeled cells were analyzed by fluorescence-activated cell sorting (FACS) using Cell Quest software (BD Biosciences). Cells were discriminated into subsets that were apoptotic (sub G0/G1 phase) or resided in G0/G1, S (actively proliferating), or G2+M phases of the cell cycle.

### Karyotype Analysis

Cells were seeded into 35 mm dishes at a density equivalent to about 50% confluence. Vinblastin sulfate was added to 4 µg/ml and the cells were incubated overnight. The cells were trypsinized, diluted into Robb's saline, centrifuged and resuspended in 3 ml 0.075 M KCl for 20 minutes. Four drops of fix (3∶1 methanol∶glacial acetic acid) was added and the cells were centrifuged, resuspended in 3 ml of fix and incubated for 10 minutes. Cells were centrifuged, resuspended in a small quantity of fix and spotted onto clean slides. Slides were viewed without mounting, or with ethanol and coverslips, by phase contrast and ≥50 mitotic spreads were scored for each line. The small 4^th^ chromosome was not scored, as it is often lost in cells in culture and/or difficult to visualize at the 40× magnification used. Wild-type cells were analyzed at passages 15–30, *Ras^V12^* cells at passages 16–47, *wts^RNAi^* cells at passages 7–17 and *Ras^V12^ wts^RNAi^* cells at passages 15–30.

### Cell Injections into Adults

Females (*ovo^D2^/+*, which have rudimentary ovaries and therefore more space in the abdomen for tumors to grow) were anaesthetized with ether and stuck by their wings to double-sided tape on a microscope slide. Tissue-culture cells were sucked into a glass needle and injected into the posterior ventral abdomen. Flies were scored for survival and photographed after injection and periodically to document dispersal of GFP positive cells.

### Western Blotting

Cellular lysates were prepared in TN1 lysis buffer containing 125 mM NaCl, 50 mM Tris (pH = 8.0), 10 mM EDTA (pH = 8.0), 10 mM Na_4_P_2_O_7_ ·10H2O, 10 mM NaF, 1% Triton X-100, 3 mM Na_3_VO_4_ supplemented with protease inhibitor cocktail Roche Diagnostics Corp. (Indianapolis, IN), centrifuged, and supernatants were used for analysis. Total protein (10 µg) was separated on polyacrylamide gels and immunoblots were incubated with antibodies directed against pan-Erk and β-tubulin (Santa Cruz Biotechnology; Santa Cruz, CA); dpErk1/2 (E10), *Drosophila*-specific phospho-Akt (Ser 505), and Akt (Cell Signaling Technology; Danvers, MA), GFP (BD Biosciences; Palo Alto, CA) and Ras (kindly provided by Marc Therrien).

### Immunostaining

Cells were grown in dishes on coverslips or in multi-well slide chambers and processed for antibody staining. Cells were washed once in 1× PBS and fixed for 20 minutes in 4% paraformaldheyde in PBS. Cells were rinsed briefly in PBS and washed three times in 1× PBS for 5 minutes. PBS+0.2% Triton X-100 (PBTX) was used to permeabilize the cells. Cells were washed three times in 1× PBS and blocked in PBS with 5% Normal Goat Serum (NGS) for 1 hour and incubated with primary antibody and 5% NGS, overnight at 4°C. Cells were washed 3 times in PBS and Rhodamine conjugated secondary antibodies (1∶200) were added and incubated for 30 mins-1 hour at room temperature. Cells were washed 3 times in 1× PBS and mounted using VectaShield (Vector Laboratories). Images were captured using a compound fluorescence microscope or a Zeiss 510 META Laser Scanning Confocal microscope. The following antibodies were used: D–E Cadherin (Rat)-1∶5 (Hybridoma Bank, Iowa), dMef2 (Rabbit) 1∶500 [21], H2 antibody (Mouse) 1∶10 [20], HRP- Jackson immunoresearch (Rhodamine conjugated) 1∶200. All the secondary antibodies were from Jackson ImmunoResearch.

### Fat Staining

Cells were rinsed in PBS followed by fixing in 4% paraformaldehyde in PBS for 20 minute at room temperature. Cells were briefly washed with PBS and stained with DAPI (Sigma; 1 mg/ml stock diluted to 1∶1000) and Nile Red solution (Sigma; 1% stock in DMSO diluted to 1∶5000) for 30 minutes at room temperature [53]. Cells were mounted and photographed using a fluorescent microscope.

### Microarray Analysis

Cells from Ras^V12^ line 11 at passage 12 were grown to 70% confluence and RNA was extracted (Qiagen RNeasy). Three samples derived from independent T-flasks were processed. Targets were generated and hybridized to DrosGenome1 Affymetrix gene chips using standard procedures (Affymetrix.com). The embryo datasets were from the Berkeley Drosophila Genome Project (ftp://ftp.fruitfly.org/pub/embryo_tc_array_data/), adult datasets were from the Gene Expression Omnibus (GEO) (GSM29178–GSM29182), CL8 cell line, wing disc and leg disc datasets were from Butchar et al. (in preparation; GEO series GSE10781), and cell line datasets were Kc [54], S2 (Ian Roberts personal communication; http://flight.licr.org) and BG2 [55]. All analyses were done using the Bioconductor suite of packages [56](www.bioconductor.org) in R (www.r-project.org). Expression values were calculated using the GC Robust Multiarray Average (GCRMA) method and statistical tests for differential expression were done using the ‘limma’ package [57]. Clustering was performed on the top 20% of genes ranked by standard deviation, using 1-correlation as the distance measure and an average linkage. For class discrimination analysis, the ‘pamr’ package was used [58].

### Quantitative PCR

*wts* mRNA expression was determined by realtime PCR using relative quantitation by the comparative *C*_T_ method [59]. One microliter of cDNA was subjected to real-time quantitative PCR using an iCycler (BioRad, USA) and Taqman^R^ gene Expression Assay (Applied Biosystems) designed for the *D. melanogaster wts* gene. An expression assay for eukaryotic 18S rRNA served as internal control. The reaction conditions were: 95°C for 10 min, followed by 40 cycles consisting of 95°C (15 s), 60°C (1 min). The level of *wts* expression was normalized to 18S levels using the formula 2^−ΔΔ*C*^T, where ΔΔ*C*_T_ = Δ*C*_T_ (sample) −Δ*C*_T_ (calibrator) and Δ*C*_T_ is the *C*_T_ of the internal control (18S) subtracted from the *C*_T_ of the target gene (wts). The calibrator used in our experiments was the control cell line wild type 2 (Wt3).

## Supporting Information

Figure S1 Giant cells expressing Myc and *Ras* ^V12^. (A–C) phase images of cells and (A′–C′) corresponding GFP images. All panels include fat body cells. (A, A′) Control cells expressing GFP (*Act5C-GAL4; UAS-GFP*) are a similar size to GFP-cells. (B, B′) Myc-expressing cells (*Act5C-GAL4; UAS-GFP, UAS-Myc*) are enlarged, due to endoreplication, compared to control cells (GFP-). (C, C′) The RasV12-expressing cell (*Act5C-GAL4; UAS-GFP, UAS- Ras* ^V12^) is greatly enlarged, due to endoreplication, compared to control cells (GFP-). (Scale bar, 50 µm.) Panels A and C also appear in Figure 2.
(0.94 MB TIF)

Figure S2 Ras and dpErk expression in *Ras* ^V12^-expressing cell lines. (A) Erk, dpErk, Ras and GFP expression levels were examined in 8 independent *Ras* ^V12^lines and the control cell line wild type 1 (wt1). The *Ras* ^V12^lines express robust and relatively similar levels of Ras and GFP, with the exception of line 13, which has low Ras levels. The level of Ras expression varied about 1.0–3.6 fold between the lines using line 1 as the baseline and excluding line 13. The control line, wt1, which does not express *Ras* ^V12^, has an undetectable level of endogenous Ras expression at this exposure. dpErk levels (normalized to total Erk) in the *Ras* ^V12^-expressing lines were between 11 and 33 fold higher than the control line (wt1). (B) Ras expression in *Ras* ^V12^ line 11 through various passages. The level of Ras expression changed only marginally over time (1–1.3 fold variation). Quantification was done using ImageQuant v5.0 (Amersham Biosciences).
(1.13 MB TIF)

Figure S3 Heat map showing *Ras* ^V12^-expressing cells have a similar expression profile to established cell lines. Array datasets were categorized as ‘adults’, ‘embryos’, ‘discs’, or ‘cell lines’. The ‘pamr’ software package was then used to choose a set of genes that best distinguished between these categories. The *Ras* ^V12^datasets were not included in this choosing step. To select genes that best discriminate between the categories, a pamr threshold of 20 was used. This yielded 66 genes with no misclassification errors. Expression values for these genes across all categorized datasets, as well as the *Ras* ^V12^cells, were plotted in the form of a heatmap. The *Ras* ^V12^cells (highlighted in yellow) cluster closely with the established cell lines and away from the other groups.
(3.46 MB TIF)

Figure S4 wts^RNAi^ reduces *wts* expression. (A) The level of wts RNA expression was determined in the 6 *Ras* ^V12^; *wts* ^RNAi^lines. The levels were reduced to between 10% and 75% of the wild-type level (wt3). There was no strict correlation between the fraction of polyploid cells in a line and the level of *wts* knockdown. The line (line 6) with the highest level of *wts* expression (75% of wild type) was 100% polyploid. However, this line is triploid, whereas, the others are diploid/tetraploid mixtures or fully tetraploid. Real time PCR with a Taqman probe was used to estimate the level of *wts* mRNA knockdown. The dsRNA region corresponds to exon 3, the taqman probe (Applied Biosystems assay Dm02153339_m1) spans exons 2–3 (and does not overlap with the region covered by the dsRNA). (B–D) *wts* ^RNAi^ expression causes tumor-like and overgrowth phenotypes *in vivo*. The *UAS-wts* ^RNAi^gene was expressed with the *engrailed-GAL4* driver (25°C), which induces expression only in posterior cells. (B) Wild-type abdomen. (C) *en-GAL4; UAS-wts* ^RNAi^ abdomen showing tumor-like outgrowths in the posterior ventral abdominal segments (arrowheads mark outgrowths in segment A2). (D) Wild-type proximal wing region. (E) *en-GAL4; UAS-wts* ^RNiA^ proximal wing region. The alula, a posterior structure, is enlarged compared with wild type (compare length of solid lines in D and E). The distal costal vein, an anterior structure, is about the same size as wild type (compare dashed lines in D and E).
(2.14 MB TIF)
