# Supplementary figures and images for: Efficient Genetic Method for Establishing Drosophila Cell Lines Unlocks the Potential to Create Lines of Specific Genotypes
Source: PLoS Genet. 2008 Aug 1;4(8):e1000142. doi: 10.1371/journal.pgen.1000142 (PMC2474701; doi:10.1371/journal.pgen.1000142)

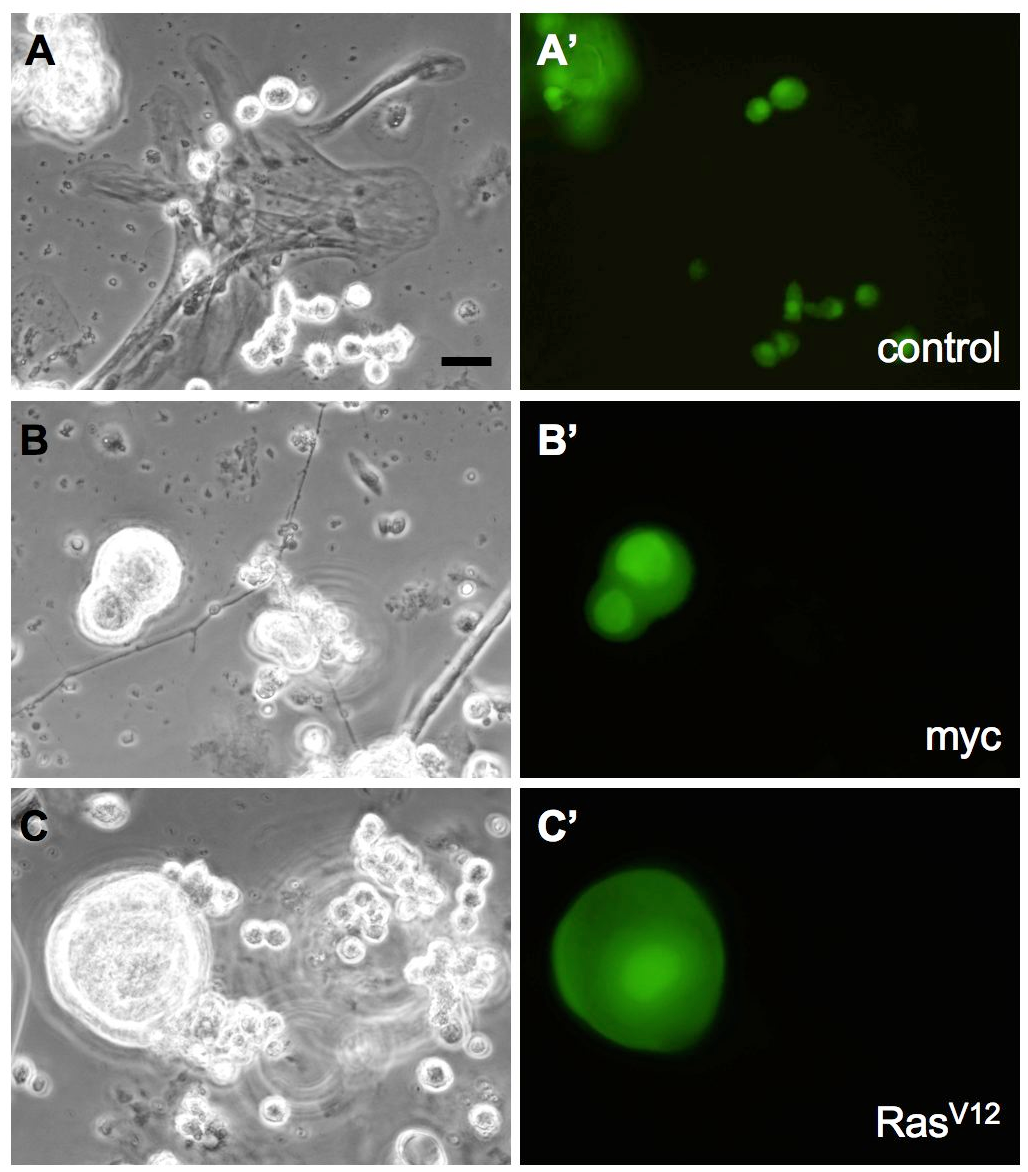

Supplement: Figure S1 — Giant cells expressing Myc and Ras V12. (A–C) phase images of cells and (A′–C′) corresponding GFP images. All panels include fat body cells. (A, A′) Control cells expressing GFP (Act5C-GAL4; UAS-GFP) are a similar size to GFP-cells. (B, B′) Myc-expressing cells (Act5C-GAL4; UAS-GFP, UAS-Myc) are enlarged, due to endoreplication, compared to control cells (GFP-). (C, C′) The RasV12-expressing cell (Act5C-GAL4; UAS-GFP, UAS- Ras V12) is greatly enlarged, due to endoreplication, compared to control cells (GFP-). (Scale bar, 50 µm.) Panels A and C also appear in Figure 2. (0.94 MB TIF) [file pgen.1000142.s001.tif]

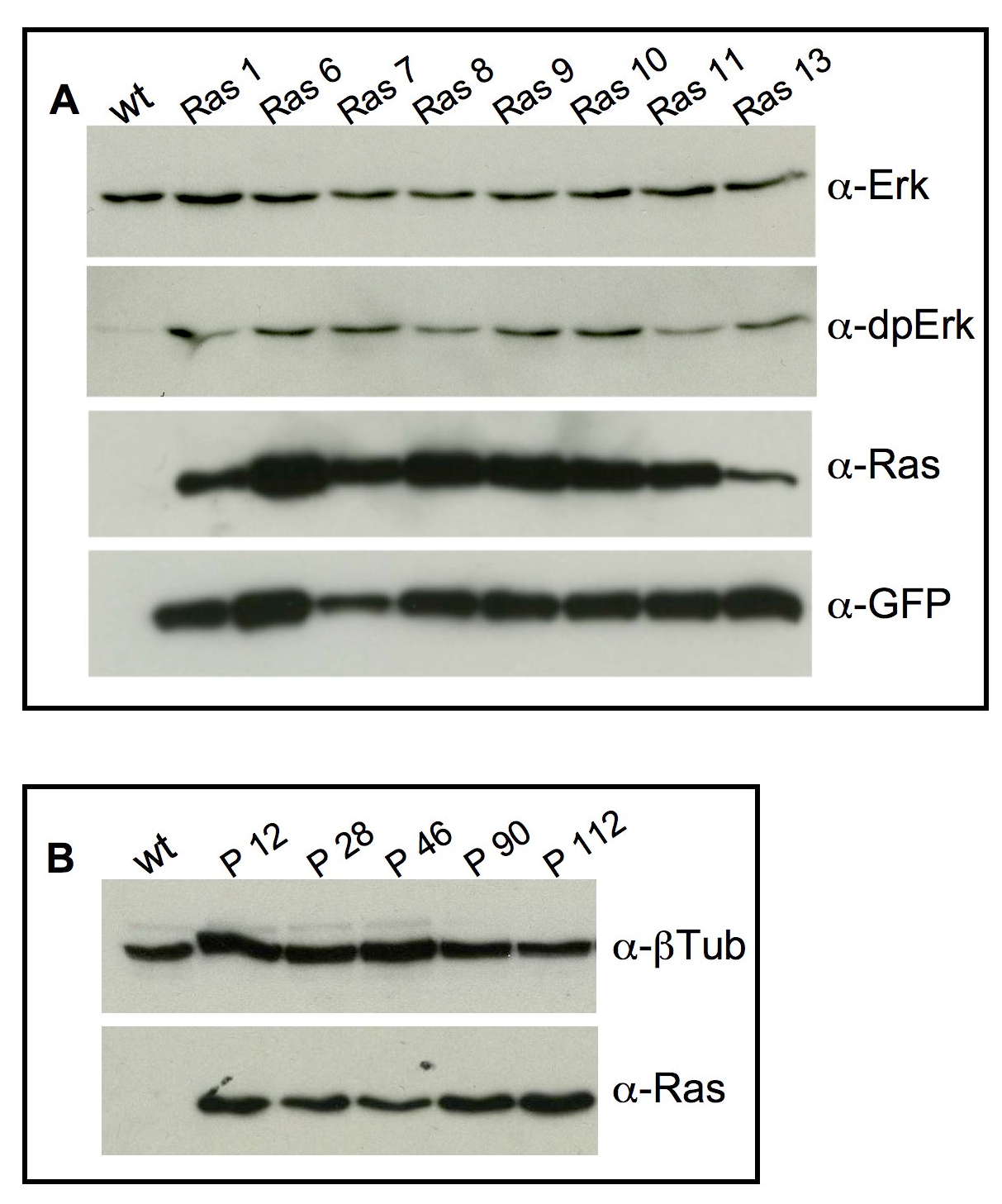

Supplement: Figure S2 — Ras and dpErk expression in Ras V12-expressing cell lines. (A) Erk, dpErk, Ras and GFP expression levels were examined in 8 independent Ras V12lines and the control cell line wild type 1 (wt1). The Ras V12lines express robust and relatively similar levels of Ras and GFP, with the exception of line 13, which has low Ras levels. The level of Ras expression varied about 1.0–3.6 fold between the lines using line 1 as the baseline and excluding line 13. The control line, wt1, which does not express Ras V12, has an undetectable level of endogenous Ras expression at this exposure. dpErk levels (normalized to total Erk) in the Ras V12-expressing lines were between 11 and 33 fold higher than the control line (wt1). (B) Ras expression in Ras V12 line 11 through various passages. The level of Ras expression changed only marginally over time (1–1.3 fold variation). Quantification was done using ImageQuant v5.0 (Amersham Biosciences). (1.13 MB TIF) [file pgen.1000142.s002.tif]

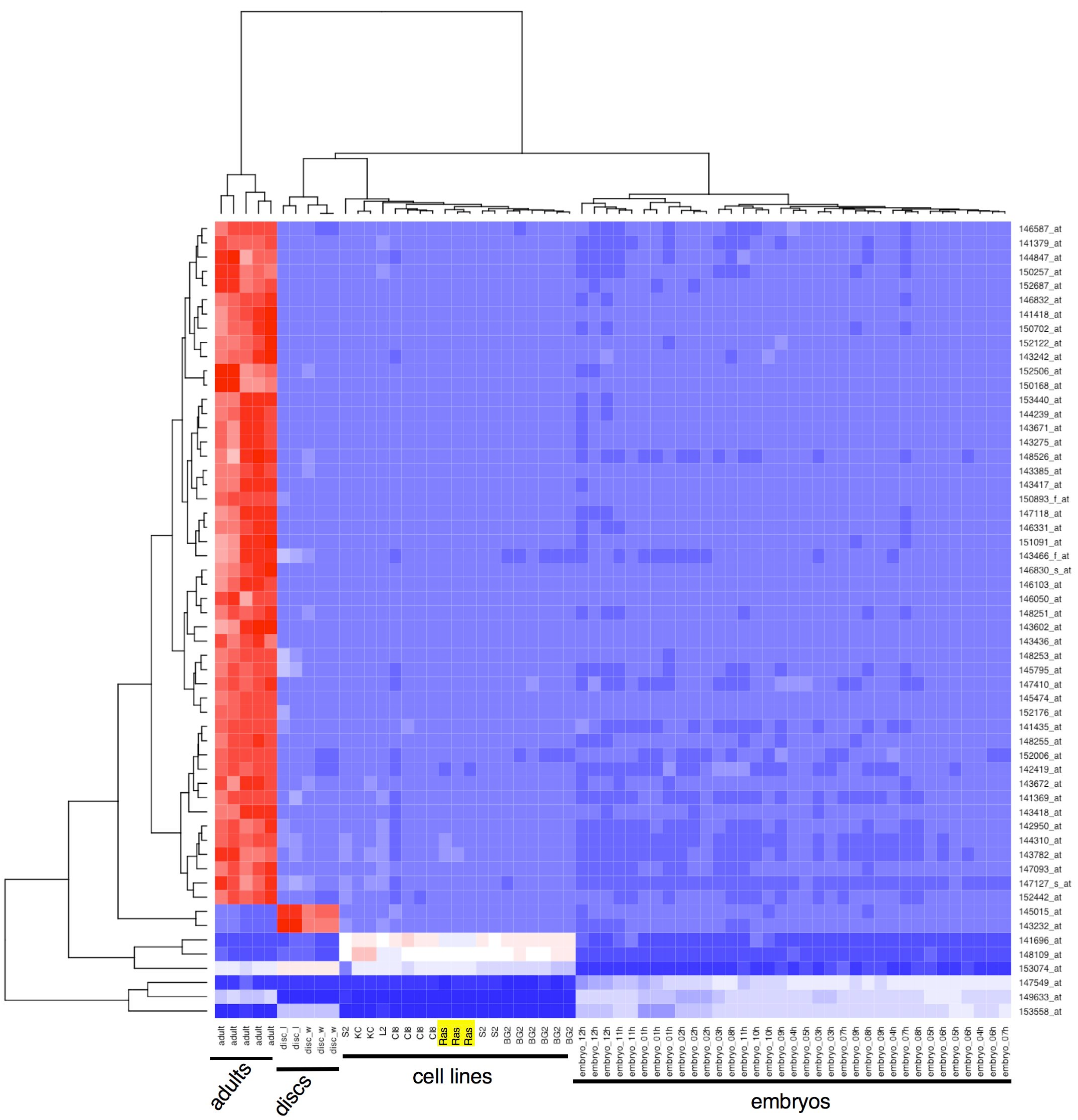

Supplement: Figure S3 — Heat map showing Ras V12-expressing cells have a similar expression profile to established cell lines. Array datasets were categorized as ‘adults’, ‘embryos’, ‘discs’, or ‘cell lines’. The ‘pamr’ software package was then used to choose a set of genes that best distinguished between these categories. The Ras V12datasets were not included in this choosing step. To select genes that best discriminate between the categories, a pamr threshold of 20 was used. This yielded 66 genes with no misclassification errors. Expression values for these genes across all categorized datasets, as well as the Ras V12cells, were plotted in the form of a heatmap. The Ras V12cells (highlighted in yellow) cluster closely with the established cell lines and away from the other groups. (3.46 MB TIF) [file pgen.1000142.s003.tif]

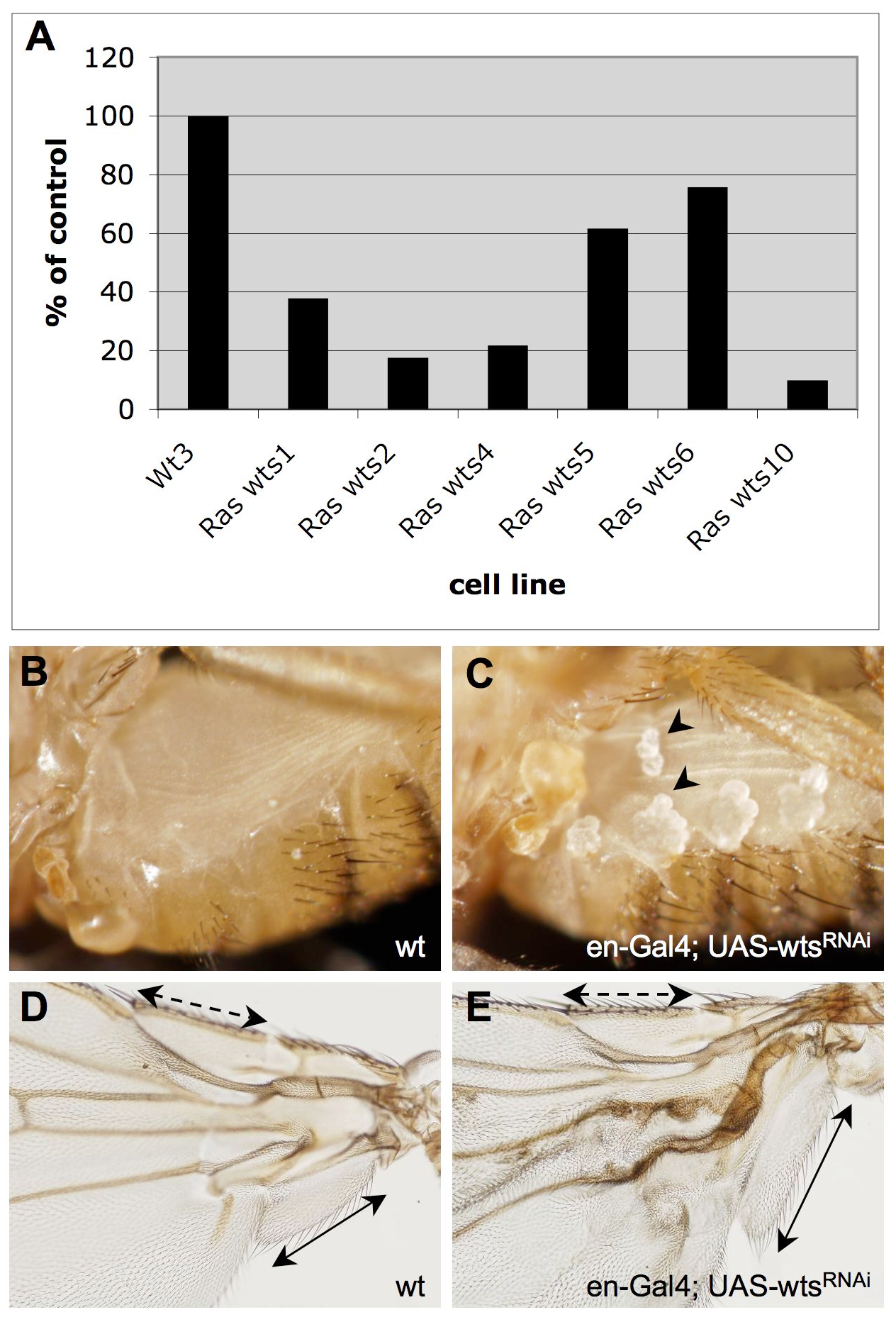

Supplement: Figure S4 — wtsRNAi reduces wts expression. (A) The level of wts RNA expression was determined in the 6 Ras V12; wts RNAilines. The levels were reduced to between 10% and 75% of the wild-type level (wt3). There was no strict correlation between the fraction of polyploid cells in a line and the level of wts knockdown. The line (line 6) with the highest level of wts expression (75% of wild type) was 100% polyploid. However, this line is triploid, whereas, the others are diploid/tetraploid mixtures or fully tetraploid. Real time PCR with a Taqman probe was used to estimate the level of wts mRNA knockdown. The dsRNA region corresponds to exon 3, the taqman probe (Applied Biosystems assay Dm02153339_m1) spans exons 2–3 (and does not overlap with the region covered by the dsRNA). (B–D) wts RNAi expression causes tumor-like and overgrowth phenotypes in vivo. The UAS-wts RNAigene was expressed with the engrailed-GAL4 driver (25°C), which induces expression only in posterior cells. (B) Wild-type abdomen. (C) en-GAL4; UAS-wts RNAi abdomen showing tumor-like outgrowths in the posterior ventral abdominal segments (arrowheads mark outgrowths in segment A2). (D) Wild-type proximal wing region. (E) en-GAL4; UAS-wts RNiA proximal wing region. The alula, a posterior structure, is enlarged compared with wild type (compare length of solid lines in D and E). The distal costal vein, an anterior structure, is about the same size as wild type (compare dashed lines in D and E). (2.14 MB TIF) [file pgen.1000142.s004.tif]
